# Technical Note: Using FIMS to determine mercury content in sewage sludge, sediment and soil samples

**DOI:** 10.1155/S1463924696000272

**Published:** 1996

**Authors:** Tiezheng Guo, Jörn Baasner

**Affiliations:** Bodenseewerk Perkin-Elmer GmbH Überlingen Germany


Journal of Automatic Chemistry, Vol. 18, No. 6 (November–December 1996) pp. 221–223

### Technical Note: Using FIMS to determine mercury content in sewage sludge, sediment and soil samples

**Tiezheng Guo and Jörn Baasner**

*Bodenseewerk Perkin-Elmer GmbH, Überlingen, Germany*

The Flow Injection Mercury System (FIMS) is an dedicated system that integrates flow injection mercury cold vapour generation with a very sensitive detector. Instrumental detection limits can be as low as 5 ng/l using a sample volume of 500 µl. The FIMS permits sample dilution, therefore reducing the likelihood of an interference occurring with complex samples.

In this study samples were digested with *aqua regia* using reflux conditions according to DIN method 38414. After proper dilution of the digested sample solution, Hg was measured interference free using $SnCl_2$ as the reductant.

The recoveries of spiked mercury in sewage sludge samples ranged from 96 to 100%. The method was checked by the analysis of standard reference materials. All results were in agreement with certified values. The RSD for three replicates was approximately 2% at 10 µg/l Hg levels.

The sample solutions were also measured using an FIAS to generate the Hg vapour and the analytical data was collected using an AA spectrometer equipped with a $D_2$ background corrector. The results are in agreement with those obtained with the FIMS, which demonstrates that any background absorption for these determinations was negligible.

### Reagents and solutions

All chemicals were at least of analytical reagent grade, and deionized water was used throughout:

(1) $SnCl_2 \cdot 2H_2O$: Pro analysi Merck.

(2) $HNO_3$: Pro analysi Merck (max. 0·000 000 5% Hg).

(3) HCl: Pro analysi Merck, 37% (max. 0·000 000 5% Hg).

(4) $K_2Cr_2O_7$: Pro analysi Merck.

Dow Corning 110 A Antifoaming reagent, Perkin-Elmer Part No. B0507226.

### *$SnCl_2$ solution*

1·5% m/v in 1% v/v HCl. 15 g $SnCl_2 \cdot 2H_2O$ were dissolved in about 150 ml water containing 10 ml HCl, and then the solution was diluted to 1 l. To 1 l of this solution 200 µl antifoaming reagent was added.

### *Carrier solution*

3% v/v HCl. 30 ml HCl was diluted to 1 l with deionized water.

### *Stabilizing solution*

0·5% m/v $K_2Cr_2O_7$ in 1 + 1 $HNO_3$ 0·5 g $K_2Cr_2O_7$ was dissolved in 50 ml of water, and 50 ml of concentration $HNO_3$ was added.

### *Calibration standards*

Stock standard mercury solution #1, 1000 mg/l, was prepared from Merk Tritisol®. Stock mercury solutions #2 and #3: 10 mg/l and 1 mg/l respectively, were prepared by further dilution of the stock standard solution #1. Calibration standards at different mercury levels were prepared from stock solutions #2 and #3 by further dilution in 3% v/v HCl. Calibration standard solutions used for the measurement of sewage samples were 0·00, 5·00, 10·00, 15·00, 20·00, and 30·00 µg/l. Calibration standard solutions used for the measurement of soil and sediment samples were 0·00, 3·00, 5·00, 7·00, and 10·00 µg/l. According to DIN 38 405-E 12, the standard solutions should contain 1% v/v of the stabilizing solution.

### Procedure

### *Sample digestion and pretreatment*

$3{\cdot}00 \pm 0{\cdot}01$ g was weighed into the digestion flask, moistened with a few drops of water, and 21 ml HCl and 7 ml $HNO_3$ were added. 10 ml $HNO_3$ was pipetted into the absorbing vessel. The digestion procedure was started under reflux conditions, also in accordance with DIN 38414, Part 12.

After cooling, the solutions were transferred from the digestion flask into a 100 ml volume flask, diluted to the volume with deionized water and mixed well. After allowing the undigested material to settle out or after filtration, 1 ml of the clear supernatant solution was placed in a 10 ml test tube, 100 µl of $K_2Cr_2O_7$ stabilizing solution was added, the mixture was diluted to 10 ml and mixed well. This solution was then ready for measurement.

For very reactive samples, the glass type gas–liquid separator should be used.

0142-0453/96 $12·00 © 1996 Perkin-Elmer

### Recovery study

A recovery study was conducted for sewage samples as follows. 1 ml of the clear supernatant solution was added to a 10 ml test tube, 100 µl of $K_2Cr_2O_7$ stabilizing solution was added, with 1 ml of 0·10 mg/l of Hg standard solution, the mixture was diluted to 10 ml and mixed. The spiked Hg concentration in the diluted solutions was 10 µg/l.

### Measured standard reference materials

To check the accuracy and reliability of this method, the following standard reference materials were used in this work:

(1) NBS 1645: river sediment.
(2) BCR No 142: light sandy soil, No. 487 (individual identification); No. 528 (individual identification).
(3) BCR No 145: trace elements in a sewage sludge, No. 310 (individual identification).
(4) BCR No 146: trace elements in a sewage sludge of mainly industrial origin, No. 265 (individual identification).

BCR No. 142, 145 and 146 are certified reference materials from the Community Bureau of Reference, Commission of the European Communities.

### Measurement with $D_2$ background corrector

Prepared solutions of the above materials were also analysed with an FIAS-400 and a Perkin-Elmer Model 4100 equipped with a $D_2$ background corrector to check for background absorption. A mercury System II EDL was used as the light source.

To learn more about the concentration levels of other coexistent metal ions in the samples, the solutions were also analysed semi-quantitatively for Cu, Ni, Pb and Zn using a Perkin-Elmer OPTIMA 3000 ICP Spectrometer.

### Results

Four sewage sludge samples and a previously-digested soil sample were analysed using the FIMS. The analytical results are listed in table 1 compared with the results obtained using the Model 4100 and $D_2$ background correction. Recoveries of spiked Hg (II) in sewage sludge sample solutions ranged from 96–100%. The values listed in the table are the mean ± standard for $N$ separate measurements.

The semi-quantitative results (µg/g) for coexisting elements in sewage sludge samples 1–4 using the Optima 3000 ICP emission spectrometer were as follows: Cu: 330–550; Ni: 35–120; Pb: 72–150; and Zn: 1100–2800. The concentrations of these constituents are similar to that found in BCR No. 145.

The measured results and the recommended values of standard reference materials are summarized in table 2.

*Table 1. Measured results of sewage sludge and soil samples.*

| Sewage sludge | FIMS (µg/g) | AAS 4100 with $D_2$ (µg/g) | Recovery (%) |
|---|---|---|---|
| 1 | 1·20 ± 0·05<br>$N = 3$ | 1·17 ± 0·04<br>$N = 2$ | 98% |
| 2 | 3·33 ± 0·05<br>$N = 3$ | 3·34 ± 0·04 | 96% |
| 3 | 3·00 ± 0·07<br>$N = 3$ | 2·96 ± 0·04<br>$N = 2$ | 96% |
| 4 | 2·09 ± 0·03<br>$N = 3$ | 2·09 ± 0·05<br>$N = 2$ | 99% |
| B 3671 (Soil)* | 297·9 ± 2·9*<br>$N = 3$ | 316·9 ± 6·8*<br>$N = 2$ | |

* This sample is a digested solution supplied by an external laboratory, the measured results represented in the solution in µg/l.

*Table 2. Measured results of reference materials.*

| Sample No. | Measured value (µg/g) | Recommended value (µg/g) |
|---|---|---|
| NBS 1645 | 1·1 ± 0·1<br>$N = 4$ | 1·1 ± 0·5 |
| BCR 142<br>No. 487 | 0·112 ± 0·007<br>$N = 5$ | 0·104 ± 0·012 |
| BCR 142<br>No. 528 | 0·113 ± 0·009<br>$N = 5$ | 0·104 ± 0·012 |
| BCR 145<br>No. 310 | 9·48 ± 0·19<br>$N = 3$ | 8·82 ± 0·88 |
| BCR 146<br>No. 265 | 9·09 ± 0·11<br>$N = 3$ | 9·49 ± 0·76 |

### Discussion

Stabilizing solution was added to the diluted sample solutions to keep the mercury content stable. It was found that the mercury content in diluted sample solution continuously decreased without the addition of the stabilizing solution.

When using $SnCl_2$ as a reductant, interferences have been reported for waters containing sulphide, chloride, copper and tellurium. Also, organic compounds which have broad band UV absorbance (around 253·7 nm) are confirmed interferences [1]. Iodide has also been reported to interfere with the measurement [2]. In general there are less severe interferences from heavy metal ions when $SnCl_2$ is used as a reductant compared to the use of $NaBH_4$ as the reductant. Proper dilution of the sample solutions can alleviate interferences.

It was found that the digested sample solution must be diluted (for example, 1 to 10) prior to measurement. Otherwise, even when 10% m/v $SnCl_2$ was used as the reductant, the measured results were low. Since the FIMS system is highly sensitive and provides improved mercury detection limits, it is possible to measure the low Hg levels even with dilution of the sample solutions.

It has been reported that there is a risk of interference from volatile nitrogen oxides when mercury is determined by FI-CVAAS in digests of samples which have been decomposed by nitric acid [3]. Concentrated *aqua regia* was used for the sample digestion. Nitrogen oxides were generated during the digestion process, especially for sewage sludge samples, because these samples contain large amounts of organic material. However, no interference or background signals were observed. The background signals of the diluted sample solutions measured with a $D_2$ background corrector were negligible.

### Conclusion

After digestion using the DIN method, the mercury levels in sediment, sewage sludge and soil samples were determined using FIMS with $SnCl_2$ as reductant. The measurement is precise, simple and fast.

Relatively high Cu, Ni, Pb and Zn contents were found in the sewage sludge samples. However, by using $SnCl_2$ as the reductant, and diluting the sample solutions, the measurements were virtually interference-free. Similarly, no interference from volatile nitrogen oxides or other nonspecific absorption signals were observed.

### References

1. EPA Method 245.1, Revision 2.3 (April 1991).
2. Welz, B. and Schubert-Jacobs, M., *Fresenius Z. Anal. Chem.*, (1988), 331.
3. Rokkjaer, I., Hoyer, B. and Jensen, N., *Interference by Volatile Nitrogen Oxides in the Determination of Mercury by Flow Injection Cold Vapor Atomic Absorption Spectrometry. Talanta*, (1993), **40**, 729–735.

